# Advances in the role of the IGF signaling system in myelodysplastic syndromes and acute myeloid leukemia

**DOI:** 10.3389/fonc.2025.1540426

**Published:** 2025-06-24

**Authors:** Yifan Wang, Xinyu Dong, Shandong Tao, Qiuni Chen, Yue Chen, Lijuan Zhang, Yuye Shi, Zhengmei He, Liang Yu, Chunling Wang

**Affiliations:** ^1^ Department of Hematology, The Affiliated Huaian No.1 People’s Hospital of Nanjing Medical University, Huai’an, China; ^2^ Northern Jiangsu Institute of Clinical Medicine, Nanjing Medical University, Huai’an, China; ^3^ Department of Hematology, Huaian Key Laboratory of Autoimmune Diseases, Huai’an, China; ^4^ Department of Hematology, The Huaian Clinical College of Xuzhou Medical University, Huai’an, China

**Keywords:** IGF-I, IGF-II, IGF receptor, IGF binding proteins, MDS, AML

## Abstract

The insulin-like growth factor (IGF) signaling system comprises functionally specific ligands (IGF-I and IGF-II), receptor (IR), and binding proteins (IGFBP). IGFs are activated by binding to their receptor, IGF-IR, which is a tyrosine kinase receptor. This activation initiates signaling cascades such as PI3K/Akt and MAPK/ErK pathways, which are essential for cell proliferation, differentiation, and survival. Growing evidence links the IGF system to various hematological disorders, yet comprehensive reviews on its role in Myelodysplastic syndrome (MDS) and acute myeloid leukemia (AML) are limited. To advance understanding in this area, we aim to summarize the emerging evidence on the involvement of IGF signaling in the pathogenesis of MDS and AML. Specifically, we highlight how dysregulation of IGF-I, IGF-IR, and IGFBPs contributes to disease progression, encompassing clonal hematopoietic abnormalities, ineffective hematopoiesis in MDS, and the development of AML. The potential therapeutic implications of targeting the IGF signaling pathway, including the role of NVP-AEW541 and NVP-ADW742 effectively suppressing AML cell proliferation and enhancing chemotherapy sensitivity, are also explored. By integrating current findings, this review provides novel insights into the mechanistic role of IGF signaling in MDS and AML and its therapeutic implications, thereby guiding future research and potential clinical applications. Given the challenges, such as pathway redundancy and therapy resistance, further investigations are necessary to validate IGF-targeted therapies and optimize their clinical utility in hematologic malignancies.

## 1 Introduction

**Figure 1:**
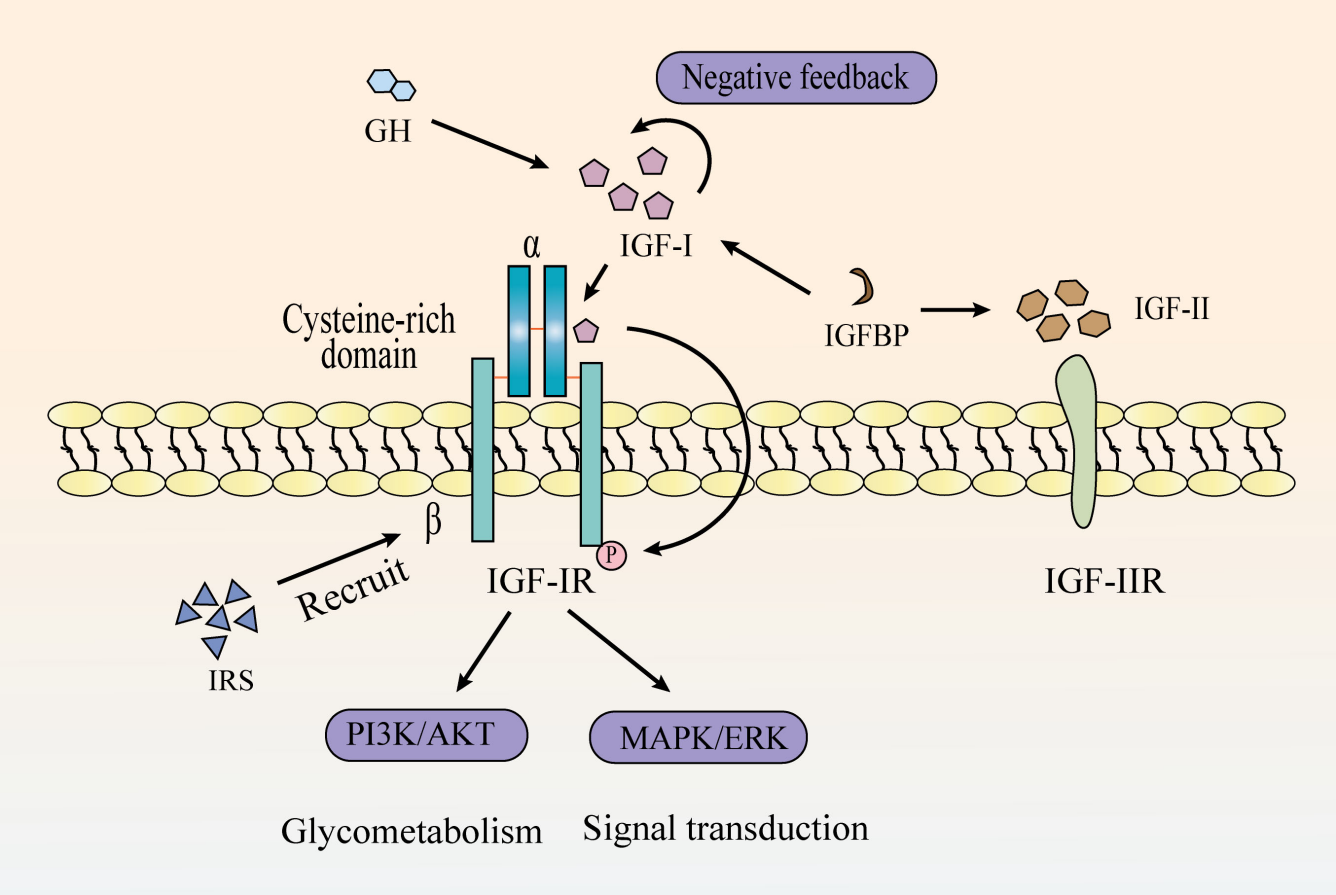
The mechanistic pathway of the IGF signaling system.

IGF system is constituted by three ligands (IGF-I, IGF-II, and insulin), four receptors (IGF type I receptor [IGF-IR]), type II receptor [IGF-IIR], insulin receptor [IR], and a heterodimeric receptor between IGF-IR and IR), and six high-affinity IGF-binding proteins (1–4) (**Figure 1**). It regulates numerous physiological processes, including cell survival, proliferation, differentiation, migration, and short-term effects like glucose uptake and metabolism (5–7). Alterations in IGF axis expression or function can lead to various pathological conditions (8, 9).

Dysregulation of the IGF axis is well documented in a variety of solid tumors, contributing to tumor progression, metastasis, and resistance to therapy (10, 11). However, growing evidence suggests that the IGF axis also plays a crucial role in hematologic malignancies, including myelodysplastic syndrome (MDS) and acute myeloid leukemia (AML). For instance, increased expression of IGF-I and IGF-IR has been observed in AML patient samples and cell lines, and correlates with enhanced PI3K/AKT pathway activation and poor clinical outcomes (12, 13). In MDS, elevated serum IGF-I levels and IGF-IR expression in CD34^+^ progenitor cells have been reported, suggesting a role in abnormal hematopoietic stem cell survival and clonal evolution (14). Moreover, bone marrow stromal cells in MDS and AML patients have been shown to secrete IGF ligands, further supporting a paracrine loop that promotes leukemic cell survival and chemoresistance (15).

MDS and AML are clonal disorders of the hematopoietic system with overlapping pathogenesis. MDS is characterized by ineffective hematopoiesis and multilineage cytopenias (16), while AML involves the rapid expansion of abnormal myeloid precursor cells that suppress normal hematopoiesis (17). MDS can progress to AML in 30%-40% of cases (18–20). The progression from MDS to AML is a multistep process characterized by the accumulation of genetic mutations and clonal selection (21, 22). Furthermore, alterations in the bone marrow microenvironment, such as elevated pro-inflammatory cytokines and dysfunctional stromal cells, also contribute to the transformation of MDS to AML (23, 24). Encouragingly, several IGF pathway–related inhibitors, such as NVP-AEW541, have shown promising preclinical activity and are being explored in early-phase clinical trials for AML (25).

Given the growing recognition of IGF signaling in hematologic malignancies, particularly in myeloid disorders such as MDS and AML, a focused review of its mechanistic relevance and therapeutic potential is timely. Although increasing attention has been paid to IGF-related pathways in these diseases, much of the current understanding is extrapolated from studies in solid tumors, and the specific roles of IGF signaling in the pathogenesis of myeloid neoplasms remain to be fully elucidated (26, 27). Therefore, this review aims to comprehensively examine the involvement of the IGF axis in MDS and AML, highlight its crosstalk with other oncogenic pathways, and summarize the current progress and challenges in targeting IGF signaling as a therapeutic strategy.

## 2 Regulation of IGF signaling system

### 2.1 IGF-I and IGF-IR

Insulin-like growth factor I (IGF-I) is a peptide hormone structurally similar to insulin but functionally distinct. Unlike insulin, which primarily regulates metabolic processes and maintains glucose homeostasis, IGF-I plays a central role in promoting cell growth and development, regulating proliferation, inhibiting apoptosis, and facilitating tissue repair (28). IGF-I mediates its biological effects mainly through autocrine and paracrine signaling. In the autocrine mode, IGF-I acts on the same cell that produces it; in the paracrine mode, it is secreted by neighboring stromal or niche cells and acts on adjacent target cells. These mechanisms are particularly critical in skeletal development and hematologic malignancies, where IGF-I contributes to cell proliferation, survival, and differentiation (29, 30). IGF-I signals through the IGF-IR receptor, activating intracellular pathways like PI3K/Akt and MAPK/Erk. These pathways are essential for cell proliferation, differentiation, and apoptosis regulation (31, 32). Upon IGF-I binding, IGF-IR undergoes conformational changes, leading to autophosphorylation of the β-subunits and recruitment of downstream signaling molecules, such as insulin receptor substrate (IRS) proteins. This activation triggers key oncogenic pathways, including the PI3K/Akt and MAPK/Erk pathways. Many researches showed that IGF-IR is highly expressed in a variety of tumor cells, and its expression level is closely related to the degree of malignancy and prognosis of a wide range of tumors (33). Given the oncogenic potential of IGF-IR, targeted therapeutic strategies have been explored, including Picropodophyllin (PPP) designed to block IGF-IR activation, has been tested in clinical trials for a range of tumors and has shown significant anti-tumor efficacy (34). The expression profiles and functional roles of IGF-IR, along with other IGF system components, are summarized in **Table 1**.

**Table 1:** Research progress of IGF signaling system related to cancer.

Target	Mechanism	Cancers	Consequence	References
IGF-I	Respond to growth hormone stimulation in the liver and generates signals through the IGF-IR	Breast cancer prostate cancer	IGF-I binding induces IGF-IR conformational changes, triggering β-subunit autophosphorylation and downstream signaling activation	(35–40)
IGF-IR	Stimulates cell proliferation, inhibits apoptosis	Primary liver cancer non-small cell lung cancer breast cancer pancreatic cancer	P53 mutation increases IGF-IR expression, while PAPP-A boosts IGF-IR activity by enhancing IGF bioavailability, worsening tumor progression	(41–44)
IGF-II	Regulate growth, fetal placental development and adult glucose metabolism	Colorectal cancer	Abnormal imprinting (LOI), which activates signaling pathways through receptors IGF-I and IR, can also lead to the expression of maternal alleles	(45, 46)
IGF-IIR	Bind to IGF-II affects the bioavailability and activity of IGF-II. Involved in the targeting and degradation of lysosomal enzymes	Liver cancer	Drives HCC malignancy by mediating MSC invasion, activating pro-cancer pathways, and sustaining cancer stem cell properties	(47, 48)
IR	Different phosphorylation states can affect the activation of downstream signaling pathways and the sensitivity of cells to insulin	Breast cancer ovarian cancer prostate cancer melanoma	Activation of PI3K/Akt and MAPK pathways promotes cancer growth, blocks cell death, and reduces sensitivity to chemo- and radiotherapy	(49–51)
IGFBPs	Regulate cell proliferation, migration, survival and autophagy	Liver cancer lung cancer breast cancer ovarian cancer colorectal cancer	Acts via IGF-independent signaling and epigenetic changes, influencing the tumor microenvironment and cell migration	(52–55)

The overexpression or activation of IGF-I signaling axis is orchestrated by multiple mechanisms, including transcriptional regulation, microRNA-mediated modulation, receptor interactions, and influences from the tumor microenvironment. Transcriptionally, the c-Myb has been shown to upregulate the expression of IGF-I and its receptor IGF-IR, thereby enhancing cellular proliferation and survival (56). At the post-transcriptional level, several microRNAs, such as miR-7, miR-99a, and miR-145, directly target the 3’ untranslated region of IGF-IR mRNA, suppressing its expression (57). Additionally, IGF-IR can form heterodimers with other receptors, like the insulin receptor isoform A (IR-A), increasing sensitivity to IGF-I and activating downstream signaling pathways that promote cell proliferation (58). The tumor microenvironment also contributes to IGF-I axis activation (59). Cancer-associated fibroblasts secrete IGF-II, which acts in a paracrine manner to activate IGF-IR signaling in adjacent tumor cells, supporting cancer stem cell properties and facilitating tumor progression (60).

At present, a number of IGF-IR-targeted pharmaceutical agents have reached the stage of clinical trials, including small molecule inhibitors and monoclonal antibodies (61). Monoclonal antibodies that target either IGF-I or IGF-II have been developed, including dusigitumab and xentuzumab. These drugs inhibit the proliferation and survival of tumor cells by inhibiting the activation of IGF-IR and blocking downstream signaling.

### 2.2 IGF-II and IGF-IIR

Similar to IGF-I, IGF-II is a single-chain polypeptide molecule comprising a specific amino acid sequence (62). IGF-II has been demonstrated to exert a substantial influence on the proliferation of tumor cells, including those associated with hepatocellular colorectal cancer (63). Notably, IGF-II is an imprinted gene, and alterations in its imprinting state, such as loss of imprinting or imprinting relaxation, have been implicated in disease progression (64). In mammals, IGF-II also plays a pivotal role in the regulation of growth, foetal placental development and glucose metabolism in adults.

The principal function of Insulin-like growth factor II receptor (IGF-IIR) is to act as a cation-independent mannose 6-phosphate receptor (65), which is involved in the targeting of lysosomal enzymes. Unlike IGF-IR, IGF-IIR lacks tyrosine kinase activity and does not directly transduce signals (66). Although IGF-IIR does not transmit signals autonomously, it is capable of influencing the bioavailability and activity of IGF-II through its binding to IGF-II. Furthermore, it is involved in the targeting and degradation of lysosomal enzymes, which is essential for maintaining normal cellular physiological functions.

### 2.3 IR

The IR is a principal transmembrane tyrosine kinase receptor (67). When insulin binds to the α-subunit of the IR, it induces conformational changes that activate the kinase domain of the β-subunit (68). The activation triggers a cascade of signaling events through the phosphorylation of substrate proteins, including members of the IRS family. The diverse phosphorylation states of IRS protein family members can influence the activation of downstream signaling pathways and cellular sensitivity to insulin (69). Aberrant IR signaling has been implicated in promoting tumor cell proliferation and invasion in cancer, while IR dysfunction has also been associated with neuronal damage and apoptosis in neurodegenerative diseases (70). This suggests that dysregulation of IR signaling may play a crucial role in both cancer progression and neurodegeneration.

### 2.4 IGFBPs

Insulin-like growth factor-binding proteins (IGFBPs) are a family of soluble proteins that regulate IGF activity by modulating its bioavailability, transport, and half-life (71). Structurally, IGFBPs consist of three main regions: the N-terminus, C-terminus, and L-region (linker region). The N-terminus and the C-terminus exhibit high conservation and are hypothesized to be associated with the high affinity of IGF. In contrast, the L-region is specific and serves as a linker between the N-terminus and the C-terminus (72).

Among the IGFBP family, IGFBP-1 has emerged as a promising serum biomarker for disease burden in various conditions. For instance, in patients with chronic hepatitis and cirrhosis, serum IGFBP-1 levels decrease while growth hormone levels increase, suggesting a feedback regulatory mechanism. In this context, hepatic resistance to GH may lead to reduced IGFBP-1 levels, which in turn affects hepatocyte repair and metabolic functions (73). Furthermore, recent studies highlight the diagnostic and prognostic utility of IGFBP-1 in cardiovascular diseases. For example, serum IGF-I and IGFBP-1 levels have been shown to correlate with different types of heart failure (74), reflecting their roles in disease progression and potential value in assessing disease burden. These findings reinforce the notion that IGFBP-1 may serve as a useful indicator of disease severity or progression in a variety of clinical settings. Additionally, IGFBP-2 has been reported to promote the proliferation and survival of hematopoietic stem cells, thereby contributing to the maintenance of hematopoietic homeostasis (75).

IGFBP-3 has been extensively studied as a biomarker for a variety of diseases. These include type I diabetes mellitus and autoimmune diseases (76). The IGFBP-4, IGFBP-5, and IGFBP-6 proteins are also important members of the IGFBPs family, which binds to IGFs and regulates their activity and distribution (77). Overexpression of Skp2B can disrupt the prohibitin-p53 axis and upregulate the expression of Pregnancy-Associated Plasma Protein A (PAPP-A) (78). PAPP-A is a metalloproteinase that enhances IGF signaling by cleaving IGFBP-4, has been implicated in various cancers (79, 80). Furthermore, a class of proteins related to IGFBPs, including IGFBP-7 to IGFBP-10, has been identified (81–84).

## 3 Cross-talk between IGF signaling and other pathways in MDS and AML pathogenesis

IGF-I binds to IGF-IR, inducing a conformational change in the receptor. The activated IGF-IR subsequently recruits PI3K, positioning it proximal to the cell membrane (85). PI3K catalyzes the conversion of phosphatidylinositol-bisphosphate to phosphatidylinositol-trisphosphate (PIP3). Accumulation of PIP3 on the cell membrane facilitates the recruitment of Akt through interaction with the PH domain of Akt via its phosphate group (86). Akt is phosphorylated and activated by phosphatidylinositol-dependent kinase 1 and mechanistic target of rapamycin complex 2 (mTORC2) while localized at the membrane (87). Following activation, Akt translocates from the cell membrane into the cytoplasm and nucleus, where it phosphorylates various downstream target proteins, including Forkhead box O (FoxO) family transcription factors and mTOR (88). Notably, IGF-I activates the PI3K/Akt pathway to enhance Mouse Double Minute 2 (MDM2), which promotes p53 degradation and supports tumor growth (89). In turn, p53 suppresses IGF-IR and induces Phosphatase and Tensin Homolog (PTEN), forming a feedback loop that regulates tumor progression (90). Interestingly, MDMX, a homolog of MDM2, is overexpressed in preleukemic states and acts as a key driver of progression to AML. Independent of p53, MDMX binds to CK1α, leading to β-Catenin accumulation and activation of Wnt/β-Catenin signaling—a non-canonical pathway through which MDMX promotes leukemogenesis (91).

In AML, Ten-Eleven Translocation 2 (TET2) mutations have been shown to enhance mTORC1 signaling, linking epigenetic dysregulation to aberrant metabolic reprogramming (92, 93). Specifically, PIK3CA mutation, PTEN mutation or inactivation, and AKT hyperactivation have been shown to lead to sustained activation of the PI3K pathway, which has been demonstrated to promote AML and MDS cell proliferation, survival, and anti-apoptosis (94–96). The TET2 mutation has been observed to be prevalent among patients diagnosed with AML and MDS (97). The occurrence of a TET2 mutation may result in the abnormal methylation of PTEN and other genes (98), this, in turn, has the potential to inhibit their normal expression and to promote excessive activation of the PI3K/AKT pathway (99). Meanwhile, activation of the PI3K/Akt/mTOR pathway contributes to chemoresistance in AML and MDS by enhancing glycolysis and lipid synthesis while suppressing autophagy, ultimately reducing chemotherapy-induced cell death (100). Importantly, this pathway also converges with the Ras/MEK/ERK axis to reinforce oncogenic signaling and reduce treatment efficacy (101). The RAS/RAF/MEK/ERK cascade is activated when growth factors like Epidermal Growth Factor or Platelet-Derived Growth Factor bind to receptor tyrosine kinases on hematopoietic or stromal cells, leading to receptor dimerization, autophosphorylation, and recruitment of adaptor proteins that activate RAS via GDP-GTP exchange (102–104). Activated RAS, in turn, engages RAF kinases, initiating a phosphorylation cascade through MEK1/2 and ERK1/2. Once activated, ERK translocates to the nucleus to regulate transcription factors such as Elk-1, Myc, and AP-1, ultimately promoting genes involved in proliferation, metabolic remodeling, and inflammation (105).

Mutations in the RAS (KRAS/NRAS) gene, found in approximately 10–30% of AML and 5–15% of MDS cases, facilitate the progression of MDS to AML and are associated with poor prognosis (106, 107). These mutations drive sustained activation of signaling pathways that promote cell cycle progression and inhibit apoptosis, leading to clonal expansion in MDS and impaired differentiation of normal hematopoietic stem cells. Additionally, ERK activation promotes AML cell proliferation, metabolic reprogramming, and secretion of pro-inflammatory cytokines, worsening bone marrow inflammation and accelerating disease progression (108). However, sustained ERK activation also enhances DNA repair and inhibits drug-induced apoptosis, thereby increasing leukemia resistance and reducing MDS treatment efficacy (109). IGF-IR activation further amplifies disease progression by engaging both the Ras/Raf/MEK/ERK and PI3K/Akt pathways, forming a dual-pathway synergy that promotes tumor cell growth and exacerbates hematologic malignancies.

The canonical Wnt/β-catenin pathway is triggered when Wnt ligands bind to the Frizzled (FZD) receptor and its co-receptors LRP5/6 on the cell surface. This interaction recruits Dishevelled proteins, which disrupt the Axin–GSK3β complex and initiate downstream signaling (110, 111). The Wnt/β-catenin and IGF signaling pathways interact through multiple mechanisms, influencing cell behavior and contributing to various physiological and pathological processes. There is significant crosstalk between these pathways; for instance, β-catenin interacts with transcription factors such as TCF/LEF and FoxO, whose activity is regulated by IGF signaling (112). Additionally, Dishevelled, a key component of Wnt signaling, can influence IGF-induced Ras-Raf-MAPK signaling (113). IGF-I also plays a crucial role in modulating the location, stability, and transcriptional activity of β-catenin (114). In the context of AML, recent studies have uncovered distinct roles of Wnt signaling (115). Notably, cytoplasmic nuclear paraspeckle assembly transcript has been found to suppress AML progression by inhibiting Wnt signaling, in contrast to its oncogenic function in other cancers (115). Furthermore, TIM-3 signaling has been shown to hijack the canonical Wnt/β-catenin pathway, thereby promoting cancer stemness in AML. Given the critical involvement of Wnt signaling in AML pathogenesis and therapy resistance, targeting the mevalonate or Wnt pathways presents a promising strategy to overcome CAR T-cell resistance in TP53-mutant AML cells (116).

Additionally, activation of the PI3K/Akt pathway has been linked to non-canonical activation of the Hedgehog (Hh) pathway, which supports leukemia cell survival and self-renewal, thereby contributing to chemoresistance in myeloid leukemia (117). Cells that secrete Hh ligands process and release these ligands, which predominantly bind in a paracrine manner to the transmembrane receptors Patched 1 (PTCH1) and PTCH2 (118). This binding inhibits the suppressive activity of PTCH1 and PTCH2 on Smoothened (SMO) (119). Subsequently, the activation of Hh signaling cascades leads to the activation and nuclear localization of GLI transcription factors, driving the expression of Hh target genes (119). Targeting this pathway has been proposed as a strategy to reduce leukemia stem cell (LSC) populations, enhance treatment responses, and improve patient outcomes (120, 121). Cholesterol directly modifies the key Hh pathway ligand, Sonic Hedgehog (SHH), enhancing its release and activation of the downstream signal molecule, SMO. This leads to upregulation of Gli1 expression, a marker of Hh signaling, promoting tumor migration and metastasis (122). P53 regulates cholesterol metabolism, influencing this pathway (123). Specifically, it has been demonstrated that the upregulation of SHH and GLI1 expression in AML cells results in aberrant activation of the Hh pathway, particularly in CD34^+^ AML cells that are resistant to chemotherapeutic agents (124). Moreover, persistent activation of SMO in mouse models and patient samples has been shown to lead to the persistence of dormant BCR-ABL+ LSCs (125). Furthermore, the use of SMO inhibitors in mouse models and human leukemia has been demonstrated to inhibit Hh signalling (126). And the Gli3 transcription factor within the Hh pathway directly regulates IGF-I expression in its activator form, while its repressor function controls IGFBP-1 levels (127). This highlights a mechanistic link between the Hh and IGF pathways, underscoring the therapeutic potential of targeting this axis. Inhibiting the Hedgehog pathway, particularly in relapsed or refractory AML, has emerged as a promising strategy (128). Glasdegib, the first and only FDA-approved Hh pathway inhibitor for AML, is used in combination with low-dose cytarabine for patients ineligible for intensive chemotherapy (129). Moreover, targeting the Hh/IGF-IR/PI3K/Akt/MRP1 axis may offer an effective therapeutic approach for refractory AML (130). However, resistance mechanisms and the need for personalized treatment strategies remain critical challenges for future research.

**Figure 2:**
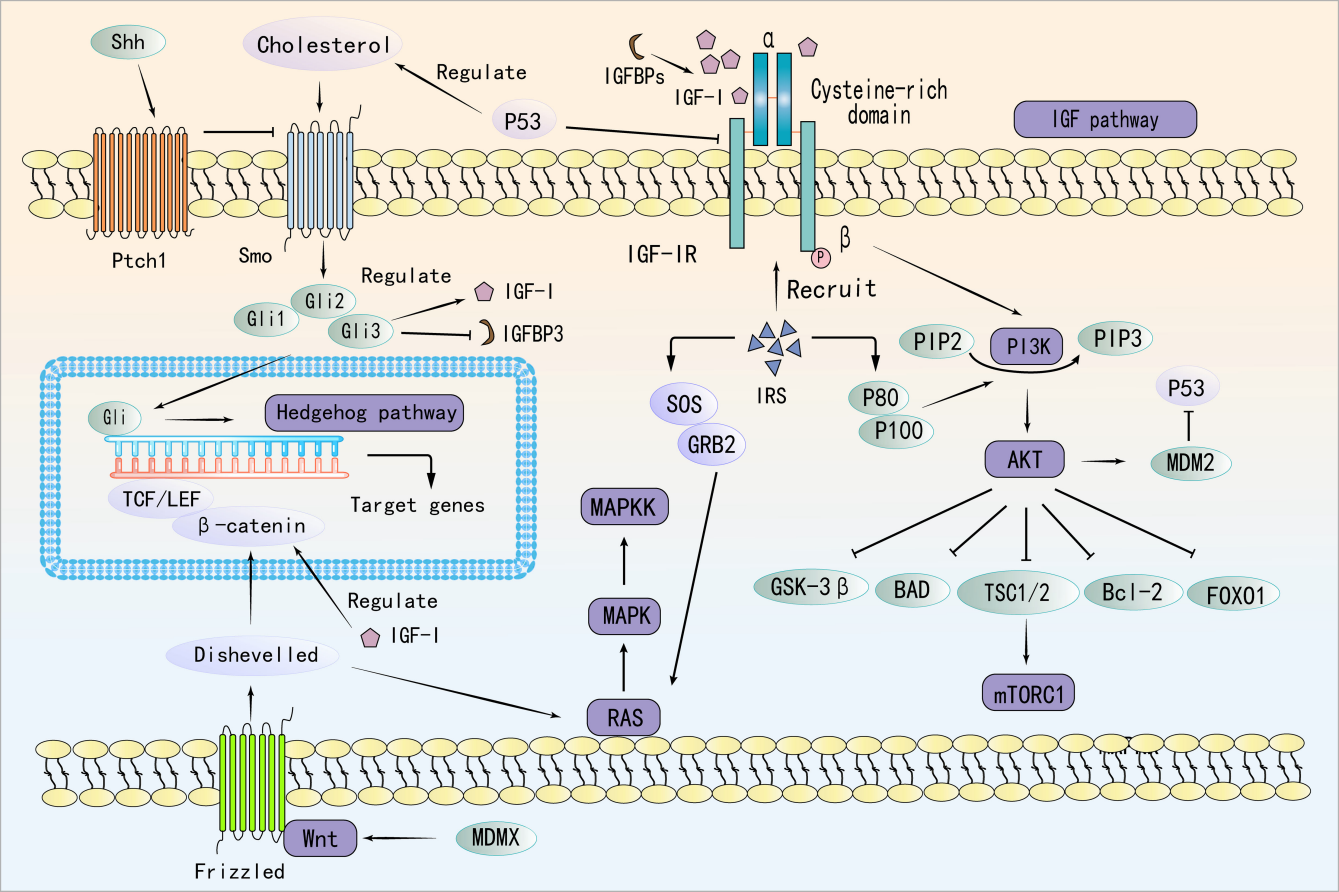
Molecular Interplay Between IGF, PI3K/Akt/mTOR, Ras/Raf/MEK/ERK, Wnt/β-Catenin, and Hedgehog Pathways.

In summary, IGF signaling interacts with multiple oncogenic pathways, including PI3K/Akt/mTOR, Ras/Raf/MEK/ERK, Wnt/β-catenin, and Hedgehog, collectively driving MDS and AML progression. These interactions contribute to enhanced proliferation, metabolic reprogramming, immune evasion, and chemoresistance. **Figure 2** provides a schematic representation of these interconnected signaling pathways. Given these intricate connections, targeting IGF signaling in combination with inhibitors of these pathways may provide a more effective therapeutic approach for MDS and AML, warranting further investigation.

## 4 IGF signaling system in the bone marrow microenvironment of MDS and AML

Unlike its role in promoting migration and invasion of MM cells (131–133), the IGF signaling pathway primarily exerts anti-apoptotic and pro-proliferative effects in MDS and AML. Comparative studies have shown significant alterations in the expression levels of certain genes within the IGF gene family in patients with AML compared to healthy bone marrow samples, with a notable increase in the expression of IGF-I, IGF-IR, and IGFBP-3 (134, 135).

Beyond its direct effects on leukemic cell proliferation and survival, IGF signaling also shapes the hematopoietic microenvironment. The bone marrow microenvironment (BMME), comprising mesenchymal stromal cells (MSCs), endothelial cells, and a spectrum of immune cells—plays a pivotal role in regulating hematopoiesis and supporting disease progression (136–138). A study found that IGF expression showed a downward trend in MDS-MSCs, suggesting that IGF dysregulation in the bone marrow niche may contribute to ineffective hematopoiesis and disease progression in MDS (139).

MSCs are central players in the BMME, and their interaction with leukemic cells is significantly influenced by IGF signaling. Bone marrow-derived MSCs have been shown to modulate the expression of ATP-binding cassette transporters in AML cells via the IGF-I pathway, thereby promoting chemotherapy resistance (15). Moreover, MSCs can secrete IGF-I to stimulate the proliferation of endothelial progenitor cells through PI3K/Akt activation, highlighting the pro-angiogenic and supportive roles of IGF signaling within the leukemia microenvironment (140).

Targeting the PI3K-Akt-mTOR axis in AML presents a particular challenge, as its dysregulation is not only driven by leukemic cell-intrinsic factors but also by cues from the microenvironment, including MSCs and immune cells (141). This complexity underscores the need to consider the BMME as an integral component of therapeutic strategies. Indeed, modulating IGF signaling has emerged as a promising approach to optimize MSC function and overcome microenvironment-induced resistance, potentially enhancing therapeutic efficacy (142).

Interestingly, studies from other tumor models support this concept. For example, human fetal MSCs inhibit liver cancer growth through secretion of IGFBPs, which suppress IGF-IR/PI3K/Akt signaling and reduce tumor proliferation (143). Similarly, M2-like tumor-associated macrophages in anaplastic thyroid carcinoma have been shown to promote tumor stemness and metastasis by secreting IGF-I/II and activating the IR-A/IGF-IR–mediated PI3K/AKT/mTOR pathway (144). These findings provide further evidence that IGF signaling within the tumor microenvironment can profoundly influence disease behavior.

Taken together, the IGF signaling axis contributes to both cell-intrinsic and microenvironmental mechanisms that drive MDS and AML progression. As research advances, targeting IGF signaling, either directly or via modulation of the BMME, may represent a cornerstone in the development of more effective treatment strategies for hematological malignancies (145).

## 5 Role of the IGF signaling system in MDS and AML

### 5.1 IGF-I in MDS and AML

IGF-I not only promotes the growth and development of the organism, but also regulates cell proliferation, differentiation and metabolism (146). In contrast to solid tumors, which have been extensively studied, relatively few studies have investigated the role of IGF-IR in leukemia, with the majority of these focusing on plasma cell myeloma (25, 147). Reduced IGF-I signaling is associated with ineffective hematopoietic features commonly observed in MDS (12). This dysregulation highlights the critical role of the IGF signaling system in maintaining hematopoietic homeostasis and its disruption in disease states (148). IGF-I exerts a significant influence on the clonal growth of hematopoietic cells in AML patients, particularly during active disease phases (13). Specifically, *in vitro* experiments confirm that IGF-I promotes the growth of AML cells primarily through the activation of the PI3K/Akt and ErK signaling pathways, a process that aligns with the fact that uncontrolled PI3K activation is present in 50% of AML cases (149, 150). AML cells can secrete IGF-I and express its receptor, IGF-IR, establishing an autocrine positive feedback loop that leads to constitutive activation of the PI3K/Akt signaling pathway. Studies have demonstrated that in approximately 70% of AML samples exhibiting PI3K activation, this persistent activation is attributable to autocrine IGF-I/IGF-IR signaling. Furthermore, treatment with neutralizing anti-IGF-IR antibodies significantly inhibits PI3K/Akt signaling and reduces the clonogenic capacity of leukemic progenitor cells (30).

IGF-I not only directly promotes cell growth but also influences the proliferation of AML cells through other mechanisms. For instance, ADAM28 degrades IGFBP-3, facilitating IGF-I-induced proliferation (151). Hematopoietic stem cell transplantation is a key therapeutic option for patients with AML (152). However, long-term survivors often face endocrine complications, which significantly impact their quality of life. Among these complications, approximately 10% involve dysfunction of the hypothalamic-pituitary-GH/IGF-I axis (153). This highlights the critical role of the IGF signaling system in post-transplant physiological regulation. Importantly, the IGF/IGF-IR axis is not only involved in endocrine homeostasis but also plays a fundamental role in regulating cancer stem cells by sustaining their stemness, survival, and proliferative capabilities (154), suggesting that targeting this axis could have dual benefits: mitigating post-transplant complications and suppressing leukemia recurrence.

### 5.2 IGF-IR in MDS and AML

IGF-IR is highly expressed and plays a key role in MDS clonal cells (155, 156). Compared with normal controls, the mean IGF-IR expression level was significantly increased in CD34^+^ cells of 100 MDS patients, suggesting its potential as a clonal cell marker for MDS (14). IGF-IR plays a dual role in MDS pathophysiology. Current studies have demonstrated that the expression rate of IGF-IR in nucleated cells from patients with MDS and AML is significantly higher than that in normal bone marrow (157). Furthermore, IGF-IR is more strongly expressed in advanced subtypes of MDS, such as refractory anemia with excess progenitor cells and its transformed form (158), which are associated with an increased proportion of blasts in the bone marrow—a critical marker indicating a heightened risk of progression from MDS to AML. IGF-IR facilitates the growth of MDS clonal cells, driving disease progression.

IGF-IR inhibits the MAPK signaling pathway, particularly p-p38 MAPK and p-p44/42 MAPK, which are critical regulators of cell proliferation and apoptosis (159). The activation of the IGF-IR pathway has been implicated in lenalidomide resistance among MDS patients. This resistance poses a significant challenge in therapeutic management and highlights the need for targeted interventions (160). A study found that IGF-IR inhibition reduces the proliferation and survival of del(5q) MDS cells both *in vitro* and *in vivo*. Furthermore, lenalidomide-resistant del(5q) MDS cells lacking TP53 or RUNX1 remain sensitive to IGF-IR inhibition. These findings suggest that targeting IGF-IR could be a promising strategy, particularly in MDS subtypes with genetic alterations that confer resistance to standard therapies (161). Knocking down IGF-IR in MDS cells results in increased phosphorylation of MAPK, reversing its inhibitory effects. Furthermore, the use of the IGF-IR inhibitor PPP effectively suppresses MDS cell proliferation and induces cell cycle arrest and apoptosis. These findings underscore the potential of IGF-IR inhibitors as therapeutic agents in MDS treatment (158).

In malignant bone marrow nucleated cells from AML patients, IGF-IR expression is as high as 92%, significantly exceeding levels found in other hematological disorders. Research indicates that IGF-IR-positive cells exhibit lower apoptosis rates compared to IGF-IR-negative cells, patients with high IGF-IR expression (>50%) demonstrate even lower rates of apoptosis, indicating a correlation between IGF-IR expression levels and resistance to apoptosis (162). In AML, miR-628 is downregulated, leading to the loss of its regulatory suppression on IGF-IR expression, thereby contributing to the upregulation of IGF-IR (163). In AML with RAS mutations, mutant RAS can upregulate the expression of IGF-IR, thereby enhancing the activity of the PI3K/Akt and MAPK signaling pathways (164). The study has demonstrated that HOXA9 directly induces IGF-IR expression, and that knockdown of HOXA9 leads to decreased IGF-IR levels, resulting in reduced leukemic cell growth and increased apoptosis (165). In certain AML cell lines, such as HL-60 and U937, the IGF-IR forms heterodimers with IR-A. These hybrid receptors enhance sensitivity to IGF-I and IGF-II, activating downstream signaling pathways like PI3K/Akt and MAPK/ERK, which promote leukemic cell proliferation (58).

IGF-IR not only plays a critical role in enhancing anti-apoptotic effects but also acts as a key regulator in inhibiting cell proliferation, particularly through its ability to activate natural killer cells, thereby suppressing the proliferation of leukemic cells (166). IGF-IR mediates growth through the PI3K/Akt/mTOR pathway and is influenced by SUMO-I modification; this pathway plays a pivotal role in the initiation and progression of AML (167). SUMOylation enhances autocrine signaling and activates downstream pathways such as PI3K/Akt, further promoting AML cell proliferation and survival. Dual inhibition of the mTOR C1 complex and the IGF-I/IGF-IR/PI3K/Akt pathway may enhance the efficacy of mTOR inhibitors in treating AML (168–170).

Collectively, these mechanisms underscore the multifaceted role of IGF-IR in AML pathogenesis and highlight potential therapeutic targets within the IGF-IR signaling axis.

### 5.3 IGF-II in AML

In leukemia cell samples from AML patients (n=32), a significant decrease in IGF-II expression was observed in both bone marrow biopsies and peripheral blood samples (171). The study demonstrates that IGF-II can induce Nanog expression through IGF-IR signaling, thereby enhancing the stem-like properties of LSCs in AML (172). The specific mechanisms of IGF-II in AML require further investigation. Studies have shown that IGF2BP-2 enhances the expression of genes associated with glutamine uptake and metabolism, thereby promoting the survival and growth of AML cells (27).

### 5.4 IGFBPs in MDS and AML

Bone marrow plasma from early-stage MDS patients exhibits significantly higher levels of IGFBP-3 compared to healthy controls. These elevated levels may contribute to the progression of the disease. IGFBP-3 promotes apoptosis in bone marrow cells, particularly under the influence of pro-apoptotic factors such as TNF-α. This apoptotic activity may exacerbate ineffective hematopoiesis and worsen cytopenias in MDS patients (173). Emerging studies suggest that adiponectin and resistin—two critical metabolic regulators—can influence the proliferation and survival of MDS cells. These effects are mediated by modulating the expression or activity of IGFBP-3. Understanding these interactions provides new insights into the metabolic regulation of MDS pathogenesis (174).

**Figure 3:**
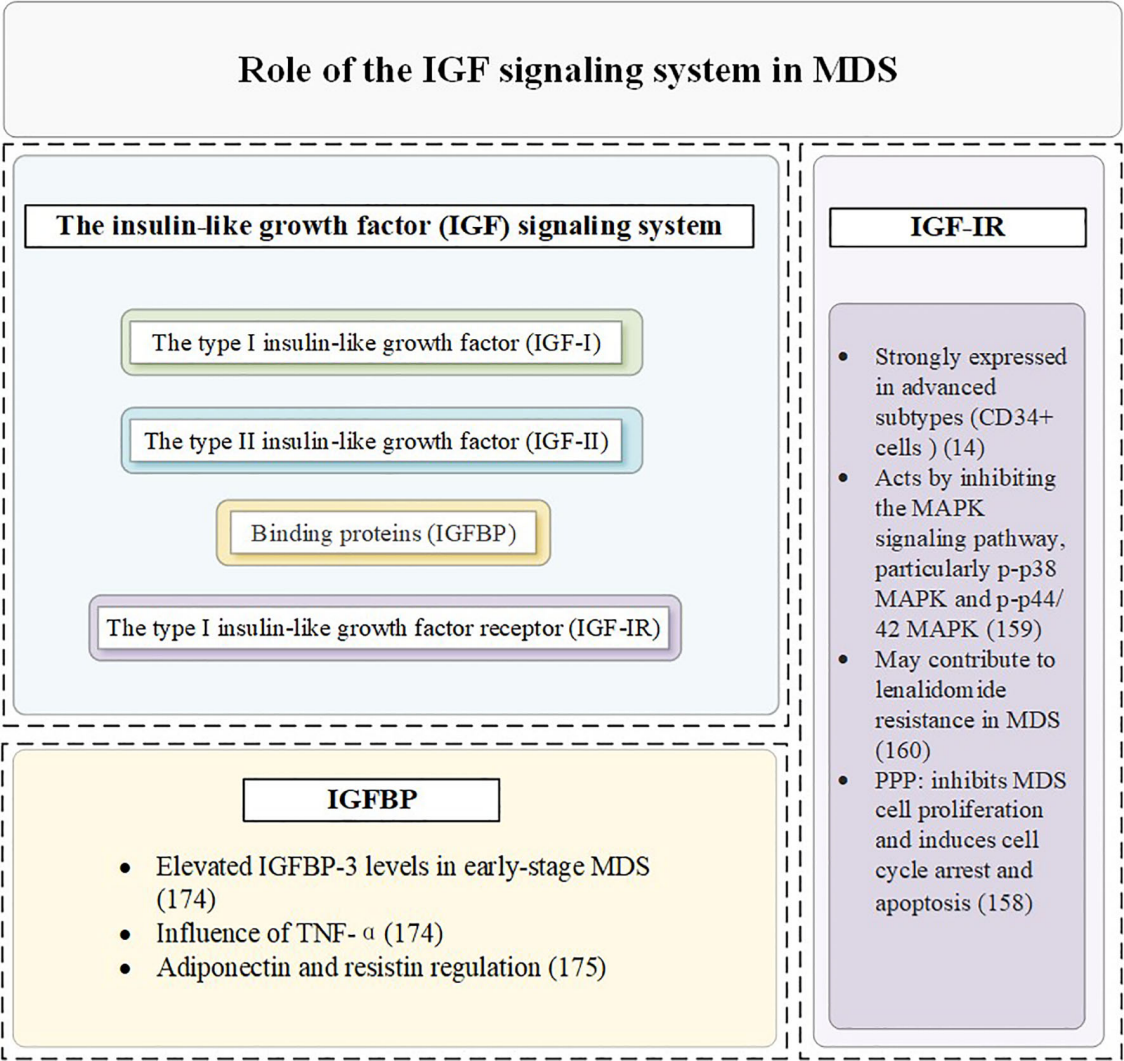
The role of the IGF signaling system in MDS.

**Figure 4:**
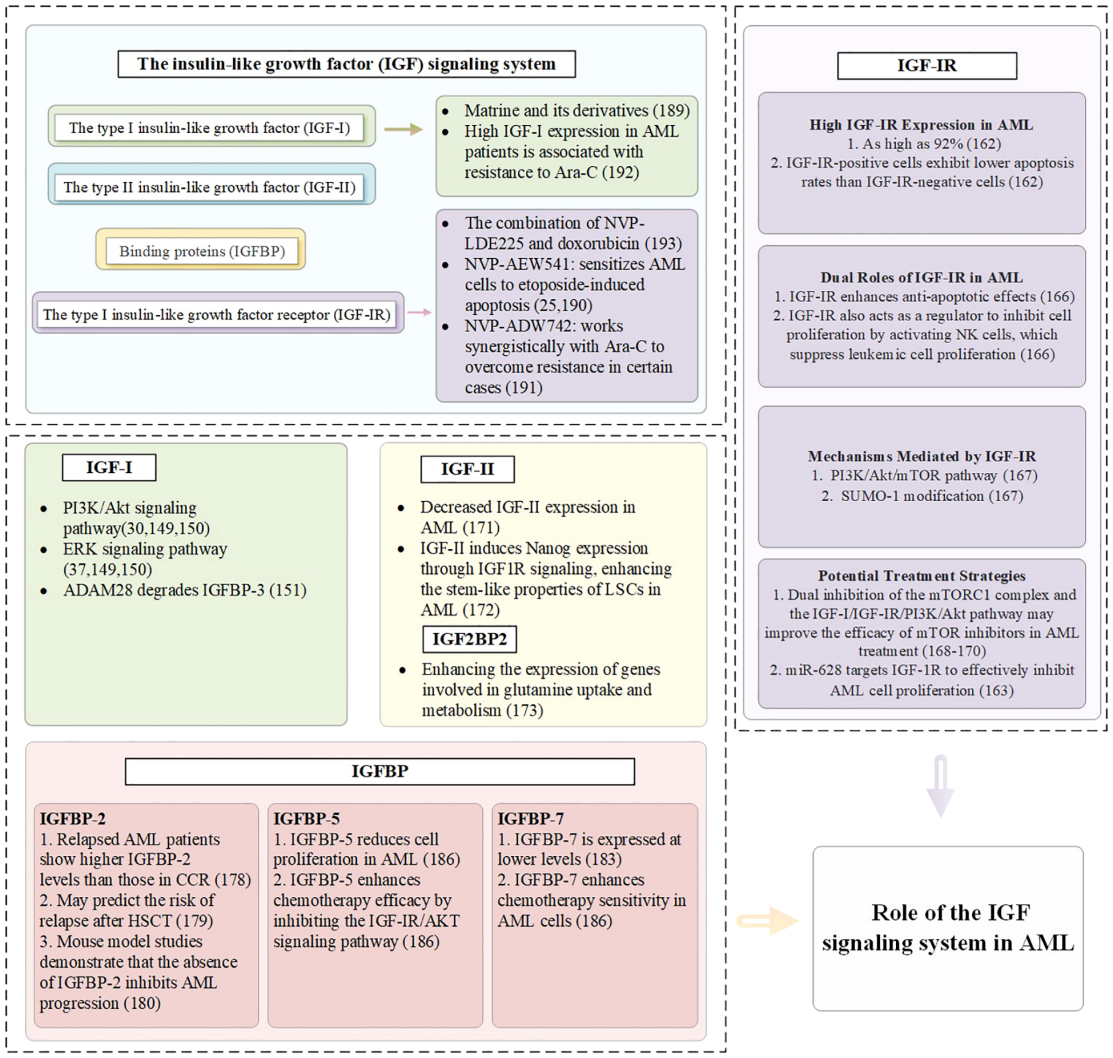
The role of the IGF signaling system in AML.

Members of the IGFBP family, particularly IGFBP-2 and IGFBP-7, play important roles in the progression of AML, treatment response, and prognostic evaluation (175). Research shows that high expression of IGFBP-2 in 99 adult AML patients is associated with the upregulation of leukemia-related genes and poor prognostic markers, as well as an increased rate of drug resistance (176). Patients with relapsed disease exhibit higher IGFBP-2 levels compared to those in complete clinical remission (177). Additionally, IGFBP-2 may indicate the risk of relapse after hematopoietic stem cell transplantation in pediatric AML patients (178). Experiments using mouse models of AML also demonstrate that the absence of IGFBP-2 can inhibit disease progression (179). In AML mouse models and human patients, elevated IGFBP-1 suppresses insulin and IGF-I activity, inducing systemic insulin resistance and supporting leukemic cell survival. Inflammatory mediators such as TNFSF13B and IL-8 may further enhance IGFBP-1 expression, aggravating metabolic dysfunction and promoting disease progression (180). In pre-treatment blood samples from non-M3 AML patients, low baseline levels of IGFBP-1 and IGFBP-6 correlate with better progression-free survival, while low baseline levels of IGFBP-2, IGFBP-2, IGFBP-6, and IGFBP-7 are closely associated with improved overall survival (181). IGFBP-7 is expressed at low levels in leukemic stem cells compared to normal hematopoietic stem cells (182). Notably, IGFBP-7 enhances the sensitivity of AML cells to chemotherapy, with higher expression levels correlating with better patient prognosis, indicating its positive role in AML treatment and outcomes (183, 184), while IGFBP-5 reduces cell proliferation and enhances chemotherapy efficacy by inhibiting the IGF-IR/AKT signaling pathway (185). The specific mechanisms of the IGF signaling pathway in MDS and AML are summarized in **Figures 3**, **4**.

### 5.5 IGF signaling pathway as a therapeutic target in MDS and AML

IGF-I reverses the antiproliferative effects of chemotherapeutic agents, such as chidamide (CHI) combined with aspirin (ASA), on AML-MDS cells (186). The combined treatment with CHI and ASA was observed to significantly down-regulate the phosphorylation levels of PI3K and AKT, thereby inhibiting the activity of the PI3K/AKT pathway. This resulted in cell cycle arrest and the induction of apoptosis. However, IGF-I, acting as an agonist of PI3K/AKT, could reverse these effects, indicating potential resistance mechanisms via IGF signaling. Despite promising preclinical results, this combination therapy has not yet advanced to clinical trials.

Additionally, a phase 1b clinical study evaluated the safety and efficacy of IGF-methotrexate (IGF-MTX) in the treatment of high-grade MDS or oligoblastic AML. While two patients demonstrated prolonged survival and reduced clonal MDS burden, the trial was limited by its small sample size, lack of a control arm, and short follow-up duration. Furthermore, the study emphasized the need for further research to better define pharmacokinetics, optimal dosing, and long-term safety (187).

The primary reason for the extremely poor prognosis in AML is the treatment failure caused by chemotherapy resistance. Matrine and related compounds may help overcome resistance mechanisms mediated by high levels of IGF-I and p-Akt activation in AML cells by inhibiting the IGF signaling pathway (188). However, current evidence for matrine’s efficacy is primarily limited to *in vitro* and animal studies. Moreover, potential toxicities, pharmacokinetics, and long-term safety of matrine in humans remain unclear. Further translational and clinical investigations are warranted to determine its therapeutic potential in AML patients.

IGF-IR inhibitors provide new strategies for the treatment of AML. The small molecule inhibitors NVP-AEW541 and NVP-ADW742 exhibit significant anti-AML activity. NVP-AEW541 inhibits AML cell proliferation and sensitizes cells to etoposide-induced apoptosis (25, 189), while NVP-ADW742 induces apoptosis in AML cells and works synergistically with Ara-C in resistant specimens (190). High IGF-I expression in AML patients correlates with Ara-C resistance, confirming the role of IGF-I in resistance mechanisms (191). In mouse models of AML, the combination of NVP-LDE225 and doxorubicin demonstrates significant anti-tumor effects, potentially related to the inhibition of the Hh/IGF-IR/Akt/MRP1 pathway (192). NVP-LDE225, an inhibitor of the Hh signaling pathway, reduces the expression of p-IGF-IR and p-Akt, which inhibits the activity of this signaling pathway and consequently decreases cell survival signaling and promotes apoptosis. Furthermore, NVP-LDE225 diminished the expression of MRP1, a drug efflux pump, and augmented the sensitivity of cells to chemotherapeutic drugs such as paclitaxel, effectively reversing the drug resistance of tumor cells. Although these findings collectively highlight the critical roles of IGF-I and its receptor in the pathogenesis and resistance mechanisms of AML, most studies remain at the preclinical stage. The clinical translation of IGF-IR inhibitors faces several anticipated challenges, including potential off-target toxicity and limited efficacy in heterogeneous patient populations. Notably, due to the structural similarity between IGF-IR and the IR, some inhibitors may inadvertently interfere with metabolic regulation, a concern raised in previous drug development efforts (193, 194). Moreover, activation of compensatory pathways such as Ras/MAPK or mTOR has been suggested to attenuate therapeutic benefit (195).

To address this, combining IGF pathway inhibitors with other molecularly targeted therapies has been suggested as a potentially promising strategy. For instance, Fms-like tyrosine kinase 3 (FLT3) mutations, particularly FLT3-ITD, are prevalent in AML and contribute to disease progression (196). Combining FLT3 inhibitors with IGF-IR inhibitors may theoretically produce synergistic effects by concurrently targeting multiple proliferative and survival pathways (197, 198). Similarly, IDH1/2 mutations, which lead to epigenetic dysregulation and metabolic reprogramming in AML, may also represent actionable targets in combination regimens (199–201). While conclusive data in AML are lacking, preliminary evidence from other malignancies raises the possibility that IGF pathway inhibition, when combined with other targeted approaches, may enhance therapeutic efficacy. Future research is warranted to evaluate such strategies in AML. Such biomarker-driven combination therapies may enhance efficacy, overcome pathway redundancy, and improve patient outcomes.

Of particular note is the recent research has combined 188Re-antiCD20 radioimmunotherapy with stable silencing of IGF-IR for the treatment of Raji cells (a model for non-Hodgkin lymphoma), demonstrating potential efficacy (202). Blocking IGF-IR signaling reduces cell proliferation and sensitizes cancer cells to ionizing radiation (203). However, the efficacy of this therapeutic approach in MDS and AML requires further investigation. Collectively, these findings highlight the critical roles of IGF-I and its receptor in the pathogenesis and resistance mechanisms of AML, suggesting that their inhibitors offer promising new avenues for AML treatment. A summary of the key therapeutic agents targeting the IGF pathway in MDS and AML is provided in **Table 2**.

**Table 2:** IGF pathway-targeting drugs in MDS and AML: mechanisms and synergistic effects.

Drug	IGF Association	Effect in AML/MDS	Drug Action	Clinical Status	Limitations	Synergistic Effects	References
NVP-AEW541	IGF-IR	AML	Inhibit AML cell proliferation	Preclinical	Off-target effects; not evaluated in clinical trials	Etoposide	(25, 189)
NVP-ADW742	IGF-IR	AML	Induce apoptosis in AML cells	Preclinical	No clinical data; unclear long-term efficacy	Ara-C	(190)
NVP-LDE225	IGF-IR	AML	Inhibit Hedgehog signaling and indirectly affects IGF-IR/Akt/MRP1 pathway	Preclinical	No human data; indirect effect on IGF-IR	Paclitaxel	(192)
Picropodophyllin	IGF-IR	MDS	Suppress MDS cell proliferation and induce cell cycle arrest and apoptosis	Preclinical	*In vitro* only; no clinical data; potential off-target toxicity and unknown pharmacodynamics *in vivo*	/	(34, 158)
Lenalidomide	IGF-IR	del (5q) MDS	IGF-IR inhibition reduces the proliferation and survival of lenalidomide-resistant del(5q) MDS cells lacking TP53 or RUNX1	Preclinical	Lack of clinical trial data, further research required	**/**	(161)
CHI-ASA	IGF-I	MDS, AML	IGF-I reverses antiproliferative effects of it by reactivating PI3K/Akt	Preclinical	Toxicity not fully studied; lacks *in vivo* validation	/	(186)
IGF-MTX	IGF-I	high-grade MDS, O-AML	Potentially safe and effective treatment	Phase 1b	Small sample size; lacks control group; early-stage	/	(187)
Matrine	IGF-I/IGF-IR	AML	Overcome the resistance mechanisms mediated by high levels of IGF-I and p-Akt activation	Preclinical	No *in vivo*/clinical data; potential off-target effects	/	(188)

## 6 Conclusion

The IGF signaling pathway plays a pivotal role in the pathogenesis and progression of MDS and AML by promoting clonal proliferation, survival, and chemotherapy resistance. Dysregulation of IGF-I, IGF-IR, and IGFBPs, particularly IGFBP-2 and IGFBP-7, has profound implications for disease progression and patient prognosis. Targeting key components of this pathway, such as IGF-IR and IGFBPs, represents a promising therapeutic strategy.

However, clinical translation has been hampered by several challenges, including pathway redundancy with IR signaling, limited efficacy in genetically heterogeneous populations, and a lack of durable responses in clinical trials. Future strategies should prioritize biomarker-driven clinical trials to identify patient subsets most likely to benefit from IGF-targeted therapies. Combination regimens that integrate IGF pathway inhibitors with agents such as FLT3 or IDH1/2 inhibitors may enhance efficacy through synergistic effects. Furthermore, rational therapeutic design must address compensatory signaling and IR redundancy, potentially through selective dual inhibitors or pathway-specific degradation approaches. Ultimately, the successful clinical application of IGF-targeted therapies will depend on addressing these challenges and validating their efficacy in large-scale clinical trials.
